# Betaine as a Functional Ingredient: Metabolism, Health-Promoting Attributes, Food Sources, Applications and Analysis Methods

**DOI:** 10.3390/molecules28124824

**Published:** 2023-06-17

**Authors:** Dejan Dobrijević, Kristian Pastor, Nataša Nastić, Fatih Özogul, Jelena Krulj, Bojana Kokić, Elena Bartkiene, João Miguel Rocha, Jovana Kojić

**Affiliations:** 1 Faculty of Medicine Novi Sad, University of Novi Sad, 21000 Novi Sad, Serbia; dejan.dobrijevic@mf.uns.ac.rs; 2 Institute for Children and Youth Health Care of Vojvodina, 21000 Novi Sad, Serbia; 3 Faculty of Technology Novi Sad, University of Novi Sad, 21000 Novi Sad, Serbia; kristian.pastor@uns.ac.rs (K.P.); natasa.nastic@uns.ac.rs (N.N.); 4 Department of Seafood Processing Technology, Faculty of Fisheries, Cukurova University, 01330 Adana, Turkey; fozogul@cu.edu.tr; 5 Institute of Food Technology (FINS), University of Novi Sad, 21000 Novi Sad, Serbia; jelena.krulj@fins.uns.ac.rs (J.K.); bojana.kokic@fins.uns.ac.rs (B.K.); jovana.kojic@fins.uns.ac.rs (J.K.); 6 Department of Food Safety and Quality, Lithuanian University of Health Sciences, 44307 Kaunas, Lithuania; elena.bartkiene@lsmuni.lt; 7 Institute of Animal Rearing Technologies, Faculty of Animal Sciences, Lithuanian University of Health Sciences, Tilzes Str. 18, 44307 Kaunas, Lithuania; 8 CBQF—Centro de Biotecnologia e Química Fina—Laboratório Associado, Escola Superior de Biotecnologia, Universidade Católica Portuguesa, Rua Diogo Botelho 1327, 4169-005 Porto, Portugal; 9 LEPABE—Laboratory for Process Engineering, Environment, Biotechnology and Energy, Faculty of Engineering, University of Porto, Rua Dr. Roberto Frias, s/n, 4200-465 Porto, Portugal; 10 ALiCE—Associate Laboratory in Chemical Engineering, Faculty of Engineering, University of Porto, Rua Dr. Roberto Frias, s/n, 4200-465 Porto, Portugal

**Keywords:** betaine, metabolic pathways, disease prevention, food sources, functional applications, extraction, detection

## Abstract

Betaine is a non-essential amino acid with proven functional properties and underutilized potential. The most common dietary sources of betaine are beets, spinach, and whole grains. Whole grains—such as quinoa, wheat and oat brans, brown rice, barley, etc.—are generally considered rich sources of betaine. This valuable compound has gained popularity as an ingredient in novel and functional foods due to the demonstrated health benefits that it may provide. This review study will provide an overview of the various natural sources of betaine, including different types of food products, and explore the potential of betaine as an innovative functional ingredient. It will thoroughly discuss its metabolic pathways and physiology, disease-preventing and health-promoting properties, and further highlight the extraction procedures and detection methods in different matrices. In addition, gaps in the existing scientific literature will be emphasized.

## 1. Introduction

**Figure 1:**
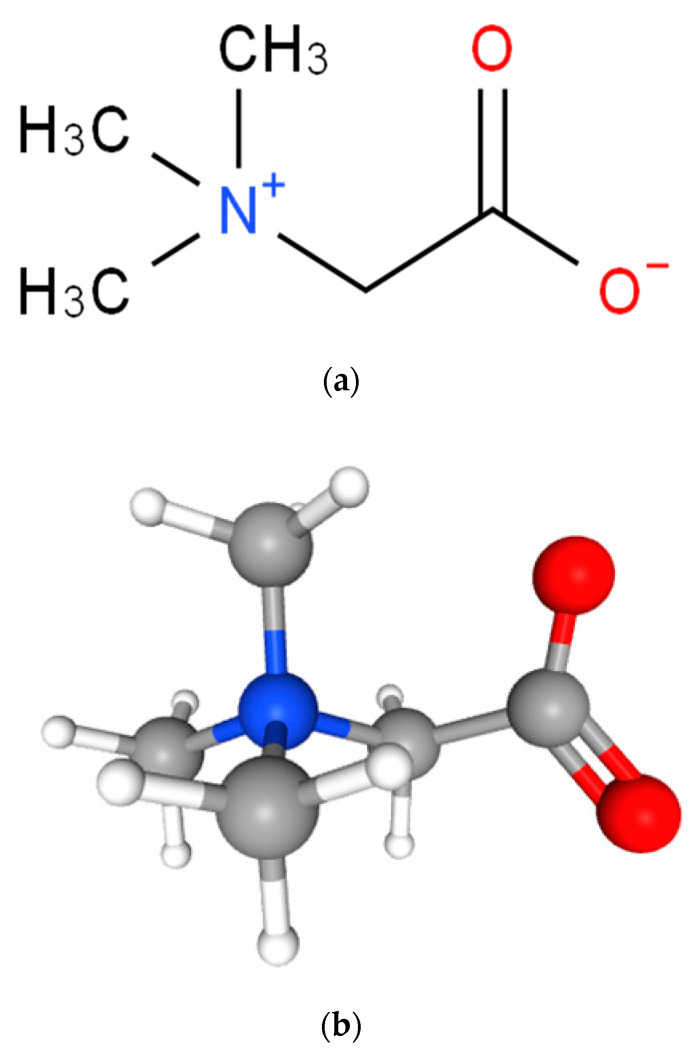
Structural formulae of betaine: (**a**) 2D-structure [5]; and (**b**) 3D-structure [6].

Betaine (trimethylglycine) is a natural product. It is a glycine derivatized by three extra methyl groups. Its chemical structural formula in 2D and 3D forms is represented in Figure 1. It is a stable and harmless natural constituent that exists in plants, animals, and microorganisms. It was reported that betaine is naturally present in beets, spinach, wheat bran, wheat germ, and aquatic invertebrates [1,2]. Betaine is endogenously produced via choline metabolism or exogenously ingested through dietary intake [3]. Either ingested as a dietary supplement or from food, betaine has a similar bioavailability, being broken down to dimethylglycine and lastly to sarcosine in the mitochondria of kidney and liver cells [4].

In the United States of America (USA), betaine is generally recognized as a safe ingredient, that is, a GRAS (Generally Recognized as Safe) ingredient, while in Europe it has approval for use in food from the European Commission (EC) [7], which allows the safe use of betaine in food in an amount of at least 500 mg per food serving. The above-mentioned health approval is associated with betaine’s contribution to the methionine cycle. Commercial betaine is available in three different forms: natural anhydrous betaine; synthetic anhydrous betaine; and betaine hydrochloride [7]. For secondary industries, betaine can be produced by chemical synthesis or by relatively expensive isolation from sugarbeets or byproducts of beet processing. Natural betaine has superior functional properties compared to its synthetic analogue, and its use is preferred by the pharmaceutical, cosmetic, and healthcare industries [8,9,10,11].

One of the main roles of betaine is to help regulate homocysteine levels in the blood [12]. Homocysteine is an amino acid that, when present in high levels, has been linked to a higher risk of heart disease, strokes, and other health problems. Betaine helps to convert homocysteine into other beneficial substances, thus helping to maintain healthy levels of this amino acid in the blood [13,14]. However, it was reported that betaine might have a harmful impact on blood lipids. Zawieja et al. [15] performed a meta-analysis on the impacts of betaine supplementation at a daily amount of at least 4 g on blood lipid status in adults. No significant impact was found for triglycerides, high-density lipoprotein (HDL) and low-density lipoprotein (LDL) cholesterol, or plasma total cholesterol. Supplementation with 4 g/d of betaine for 6 weeks could relatively increase plasma total cholesterol, which is important for cardiovascular health. Ashtary-Larky et al. [16] also carried out a meta-analysis on the impacts of betaine supplementation on cardiovascular illness indicators. They reported that supplementation with betaine at doses of 4 g/d might cause harmful effects on the lipid profiles of people with health disorders. On the other hand, supplementation with less than 4 g/d of betaine generated beneficial reductions in homocysteine levels. These findings indicate that a desirable quantity of betaine supplementation is below 4 g/d.

Betaine has also been shown to have potential benefits for exercise performance and muscle strength. Some studies have suggested that betaine supplementation may improve endurance, reduce fatigue, and enhance muscle power and strength [17]. In addition, betaine has been investigated for its potential role in supporting liver function and protecting against liver damage, as well as for its anti-inflammatory and antioxidant properties [13,18]. Overall, betaine is a compound with a range of potential health benefits, although further research is required to fully understand its mechanisms of action and effectiveness in various contexts.

Furthermore, betaine has been employed as a feed additive in pig and poultry rations since it is regarded as a cheap alternative feed supplement for improving nutrient utilization [19]. Betaine is generated by choline oxidation or delivered through diet in the animal organism. Over the past years, many studies have evaluated the impacts of betaine on various animal species. Betaine plays a role in the transmethylation reaction for the synthesis of some active constituents, such as carnitine, creatine, etc., as well as in the higher usage of nutrients and the digestibility and bioavailability of methionine. Enhancing immune status and decreasing heat or oxidative stress are also considered significant functions of betaine [20]. Most of the published research has been focused on the growth-stimulating, higher milk-yielding, carcass-modifying, immune-boosting, and stress-dropping properties of betaine in various species [21,22,23].

In addition to its potential health benefits for both humans and animals, betaine is also used in some industrial applications as a stabilizer, emulsifier, and surfactant [24,25,26]. This review will give up-to-date information on the chemical structure, physiology, and metabolism of betaine, its health-promoting and disease-preventing properties, and its functional applications.

## 2. Metabolism and Physiology

In mammals, betaine plays three important metabolic and physiological roles: (i) it is an organic osmolyte, which helps to maintain normal cell volume under osmotic stress and can accumulate to molar concentrations; (ii) it provides protection against protein denaturation, thus being called a ‘chemical chaperone’; and (iii) besides methylfolate, it is the only molecule that provides methyl groups for homocysteine remethylation [27,28,29,30].

Besides mammals, cellular uptake of betaine occurs in various other organisms, such as bacteria and invertebrates. Belonging to the chemical class of amino acids, betaine is mainly transported with the aid of γ-aminobutyric acid [31]. Studies performed on animals have shown rapid absorption of betaine after a meal in the small intestine via the duodenum [32]. Studies on humans have shown rapid absorption and distribution of betaine within 1–2 h after ingestion. The constant concentration of betaine in human serum ranges from 20 to 70 µmol/L. According to Schwahn et al. [30], rapid absorption and distribution of betaine were found both in healthy subjects and patients with homocystinuria, with a maximum concentration of 0.94 mmol/L after 0.90 h and an elimination half-life of 14.38 h. Distribution and elimination kinetics in homocystinuric patients appear to be accelerated. Even at high doses of 100 mg per kg of human weight, betaine is mostly used up through metabolic pathways and not excretion. However, betaine can be present in the urine of individuals with kidney disorders and diabetes. Subacute studies in rats have shown that betaine is not toxic when added at 0–5% of the total diet. However, due to large intakes, the ratio of red blood cells could be slightly disturbed. Many authors suggest that the maximum daily intake of betaine is 9–15 g, with 20 g being the maximum [30,33,34].

The fundamental role of betaine in microorganisms and plant cells is cell protection from possible inactivation, which may occur due to osmotic stress [35]. Exposure of plants to hostile environmental conditions, such as drought, high salinity, and unfavorable temperatures, leads to increased betaine synthesis in cell mitochondria [36]. Betaine can fulfill its role as an osmoregulator only when it is not catabolized. Modulation of water content and cell volume is of crucial importance for living organisms. Cells have the ability to adapt to varying degrees of osmotic pressure by accumulating inorganic ions of low molecular weight (such as sodium, potassium, and chlorine ions) and organic osmoregulators (such as methylamines, amino acids, and sugar alcohols). The role of inorganic ions in osmoregulation is limited because their higher concentrations could affect protein structure and thus enzyme functions [37,38]. Betaine’s osmolytic action is the result of a dipolar structure and a good solubility in water, whereby, along with the other organic osmolytes, it demonstrates a lower degree of interaction with enzyme functions and metabolic processes in cells [39]. Studies have shown that betaine has a very low potential to bind to the protein surface, thereby allowing cells to control the water surface tension without affecting lipase stabilization [40]. A cytosolic methyltransferase enzyme, betaine-homocysteine S-methyltransferase, which uses betaine as the methyl donor for the remethylation of homocysteine to form methionine and dimethylglycine, is likely to help keep the cellular osmotic equilibrium by maintaining the regular betaine concentration. Its inhibition reduces betaine degradation [30,41].

Betaine plays its primary role as an osmolyte in the kidney tissue, thus protecting the mammalian kidney medulla cells from osmotic stress and enabling the control of the concentration gradient and the accumulation of metabolic waste products in the urine [30,36,42]. It can accumulate in the kidneys of humans, protecting the cells from the high concentration of electrolytes and urea. Furthermore, it can regulate the water balance and movement through the epithelial tissue [43]. Thus, betaine increases water retention in cells by replacing inorganic salts and protecting intracellular enzymes from osmotic pressure or temperature-induced inactivation [41,44]. For example, when spinach is grown on soil with high salinity, betaine accumulates in the chloroplast and prevents water from leaking out of the cells due to the increased osmotic pressure. Mitochondria in salmon liver cells adsorb betaine when exposed to increased osmotic pressure, thus allowing the body to use less energy to maintain the required amount of water in cells. For this reason, betaine is being added as an osmoregulator to ponds to protect fish when moving through water of different salinity degrees [36].

As already mentioned above, betaine plays its second major role in methionine metabolism by converting and detoxifying homocysteine to methionine in the human liver and kidneys, acting as a methyl group donor [45,46]. Even a slightly elevated level of homocysteine in the blood could be considered a biomarker of increased risk of cardiovascular, cerebral, and peripheral vascular diseases, neurodegenerative disorders, and cognitive decline [47,48]. Moreover, higher homocysteine concentrations have been associated with low concentrations of B vitamins, such as folate and vitamins B-12 and B-6, which are involved with a one-carbon metabolism, pointing to its disturbance [49]. Research has proven that betaine intake can contribute to the lowering of circulating homocysteine levels in patients with homocystinuria and chronic renal failure, but even in healthy subjects [50,51]. The association between dietary intakes of betaine and the concentration of homocysteine has been assessed by Chiuve et al. [52]. A cross-sectional analysis of 1477 women proved that total betaine intake, together with its precursor choline, was inversely associated with plasma homocysteine levels. Furthermore, the authors concluded that the remethylation of homocysteine may be more dependent on the betaine pathway when other methyl sources are low, which might be a result of either inadequate folate intake or heavier alcohol consumption [52]. According to the study of Lee et al. [49], performed on food-frequency questionnaires on 1325 male and 1407 female participants in the USA, a higher choline-plus-betaine intake managed to decrease the concentration of post-methionine-load homocysteine. Betaine and choline intakes were, therefore, associated with both fasting and post-methionine-load total homocysteine concentrations, especially in participants with low folate and vitamin B-12 status [49]. Since betaine is found in high amounts in wheat aleurone, research by Price et al. [42] investigated the impact of a diet rich in whole-grain foods on 79 healthy participants over four weeks. The results showed a significant increase in plasma betaine concentrations, as well as dimethylglycine and methionine as products of betaine-mediated homocysteine remethylation, and a significant decrease in plasma homocysteine and LDL cholesterol levels. No significant effects on plasma choline or B vitamins (folate, riboflavin, and vitamin B-6) were thereby observed [42].

**Figure 2:**
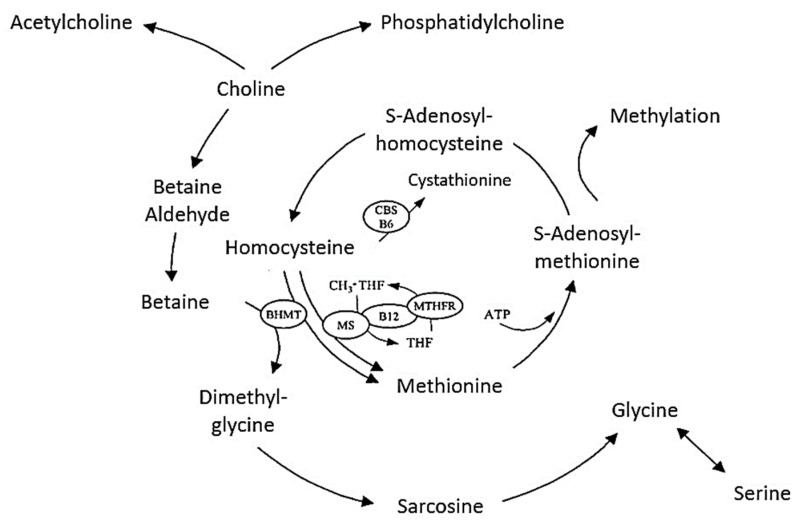
Methionine metabolic pathway [53].

Betaine could be produced in the human body by a series of enzymatic reactions that mainly occur in the mitochondrial cells of the liver and kidneys. These transmethylation reactions imply the transfer of methyl groups via the methionine cycle in vital biological processes, as shown in Figure 2 [53].

The transfer of a methyl group from betaine is conducted via the enzyme betaine-homocysteine methyltransferase (BHMT), whereby betaine is converted to dimethylglycine. In this metabolic pathway, methionine is formed from homocysteine. Alternatively, methionine could be created from 5-methyltetrahydrofolate (CH_3_-THF), which uses the enzyme methionine synthetase (MS) to transfer a methyl group via vitamin B-12 (cobalamin). The resulting compound—methylcobalamin, then donates a methyl group to homocysteine, thus forming methionine. The enzyme methylenetetrahydrofolate reductase (MTHFR) is involved in the release of the methyl group from CH_3_-THF (5-methyltetrahydrofolate). Transmethylation metabolic pathways closely link choline, betaine, methionine, CH_3_-THF, and vitamins B-6 and B-12 in order to convert homocysteine to methionine (Figure 2). This cycle also creates S-adenosyl methionine (SAM), which is considered the main methyl group donor in the human body. Moreover, SAM is involved with many other metabolic processes, such as the synthesis of deoxyribonucleic acid (DNA) and ribonucleic acid (RNA), carnitine, creatine, neurotransmitters, phospholipids, and the process of tissue regeneration, to name a few [30,45,54].

Dietary betaine, as well as L-carnitine and choline, which can be found in red meat, dairy products, chicken, eggs, and fish, have the ability to be broken down to trimethylamine (TMA) in the gut, which is absorbed and converted to trimethylamine N-oxide (TMAO) in the liver via the enzyme flavin-containing monooxygenase-3 (FMO3). The metabolite TMAO gained interest as being present in the highest concentrations in the tissues of the Greenland shark, which holds the distinction of being the longest-living vertebrate in the world [55]. This compound has been associated with several chronic disorders in humans, although the mechanisms of action are still not well understood [56]. A multiethnic cohort study by Fu et al. [56] aimed to investigate associations of TMAO and its precursors (betaine, choline, and carnitine) with inflammatory and cardiometabolic risk biomarkers in 1653 participants, ranging in age from 60 to 77 years old. Higher concentrations of TMAO and carnitine and lower concentrations of betaine were shown to be related to greater insulin resistance. In general, plasma TMAO concentrations were associated with a number of trimethylamine-producing bacterial taxa and, along with their precursors, may contribute to inflammatory and cardiometabolic risk pathways [56].

## 3. Dietary Sources

The human body is supplied with betaine through food that contains either betaine or choline. Since betaine can be irreversibly synthesized in the human body from free choline with the help of the choline dehydrogenase enzyme, it is not considered an essential nutrient [45]. However, the endogenous synthesis of betaine is generally insufficient to meet daily needs. Thus, betaine intake through food can be considered obligatory. In a typical Western diet, there are only a couple of food products that can be considered rich sources of betaine by containing more than 150 µg/g dry weight. According to Table 1, which lists betaine contents through various food matrices, the following could be considered rich sources of betaine: cereals and cereal grain products, pseudocereals, some vegetables, and the majority of seafood. The highest concentrations were observed in common wheat (490–1320), spelt (1296–1442), oat (200–1000), rye (444–2213), and triticale (986–1030) grains; amaranth (646–7420) and quinoa grains (610–6300); wholegrain flour (120–1503), bread (499–1000), pasta (375–1327), and couscous (544–1299); breakfast cereals (10–1041); spinach (675–1100); beetroot (750–3337); and mussels (1120–11,600), oysters (2780–2810), clams (2500), and scallops (640–1180), all expressed in µg per g of dry product weight [57,58,59,60,61,62,63].

Cereals and cereal products are the main sources of betaine in human nutrition. The records show the different contents of betaine in various cereal grains and products thereof (Table 1). Ross et al. [62] found two–four times higher betaine contents in wholegrain products compared to refined grain products. Slow et al. [63] indicated that the level of betaine depends on the level of stress during crop cultivation. For example, drought may result in higher betaine levels compared to well-watered crops. Logically, different cultivars contain different amounts of betaine [64]. Table 1 also shows the different betaine contents in cereal-based products recorded by different authors. Furthermore, it can be seen that the betaine content in cooked pasta is lower compared to uncooked pasta because soluble betaine easily dissolves in the cooking water. De Zwart et al. [60] determined that betaine loss during cooking can be between 60 and 80%.

Non-gluten food products, such as rice and corn, are generally low in betaine [61,62], exhibiting levels lower than 150 µg/g of betaine in the majority of commercially available gluten-free products.

## 4. Health-Promoting Properties

### 4.1. Redox Potential

The mechanism of betaine’s antioxidant activity is still not entirely clear. However, it could probably act dually: directly, as a “scavenger” of reactive oxygen species (ROS), and indirectly, by improving the activity of enzymatic antioxidants, e.g., superoxide dismutase (SOD) [65].

The ability of betaine to scavenge ROS has not been determined so far by classical chemical assays, such as the ferric-reducing antioxidant power (FRAP) test. On the other hand, betaine’s antioxidant activity has been determined in animal and plant models, suggesting that the interaction of betaine with the organism could be essential for its redox activity [66].

The indirect antioxidant activity of betaine is reflected in its ability to regulate sulfur-containing amino acid (SCAA) metabolism. SCAAs play a pivotal role in the synthesis of several intracellular antioxidants, such as glutathione. Betaine can increase intracellular levels of SCAA by increasing the level of methionine [67].

### 4.2. Liver Diseases

#### 4.2.1. Nonalcoholic Fatty Liver Disease (NAFLD)

NAFLD is a condition of fat accumulation in the liver without the presence of excessive alcohol consumption or other specific causes of hepatic steatosis. Although NAFLD was previously considered a relatively benign condition, numerous studies have identified the potential for progression to cirrhosis and hepatocellular carcinoma. Betaine, as a lipotrope, can reduce the accumulation of fat in the liver by increasing the oxidation of free fatty acids and reducing lipogenesis, and it additionally shows an anti-inflammatory effect [68]. Vesković et al. [69] presented a mouse NAFLD model induced by a methionine- and choline-deficient diet. In the study, the betaine-treated group showed alleviation of inflammation and steatosis, a decrease in enlarged mitochondria, and an increase in the number of autophagosomes. Additionally, betaine supplementation is important for prevention. It is known that a high-fat diet leads to a decrease in the concentration of S-adenosylmethionine. Deminice et al. [70] demonstrated that betaine supplementation in rats leads to a fourfold increase in S-adenosylmethionine and, thus, prevents the occurrence of fatty liver and liver damage. Despite numerous preclinical studies that claim a positive effect of betaine on the course of liver diseases, there is not enough data from large randomized studies to support its safe application for the clinical treatment of NAFLD [71].

#### 4.2.2. Alcoholic Liver Disease (ALD)

ALD refers to liver damage caused by chronic, excessive alcohol consumption. Alcohol cannot be deposited in the body and is therefore oxidized in the liver, which is the primary place of alcohol metabolism. During the breakdown of ethanol (alcohol in alcoholic beverages), highly toxic chemical compounds such as acetaldehyde are created, which trigger inflammation and, ultimately, the destruction of hepatocytes. Betaine supplementation can prevent alcohol-induced depletion of glutathione and cysteine in hepatocytes and, thereby, improve the antioxidant protection of these cells [72]. Li et al. [73] demonstrated that a diet enriched with betaine can reduce blood alcohol levels in rats continuously fed with alcohol. In a study conducted by Rajdl et al. [74], 117 male volunteers drank 375 mL of white wine per day for a month. In the group that consumed betaine along with wine, it was shown that this amino acid can reduce the adverse effects of moderate alcohol consumption by reducing homocysteine levels. Additionally, a study by Shen et al. [75] suggests that aberrant DNA methylation is associated with the pathogenesis of ALD, i.e., prolonged alcohol consumption may lead to DNA hypomethylation. Therefore, betaine, as a methyl donor, could have significant preventive properties in ALD.

#### 4.2.3. Other Liver Diseases

Betaine could also have beneficial effects on other hepatic diseases, such as drug-induced liver injury and hepatitis B and C infections [76]. Zhai et al. [77] reported that betaine has significant hepatoprotective effects in a rat model where liver injury was induced by carbon tetrachloride (CCl_4_). The results showed that betaine pretreatment significantly reduced levels of hepatic transaminases as well as hepatic levels of malondialdehyde. Additionally, levels of glutathione peroxidase and SOD were significantly increased. In a study conducted by Nezgoda et al. [78], 41 children with chronic hepatitis B infection in remission of acute lymphoblastic leukemia were treated with a betaine–arginine complex. Namely, the authors reported a significant reduction in pain syndromes, hepatomegaly, and the activity of hepatic aminotransferases. Another hepatotropic virus, the hepatitis C virus (HCV), is an important cause of chronic liver diseases worldwide. Interferons type I and II signaling is crucial in activating anti-viral genes, which can be suppressed by HCV. Betaine might have an important role in modulating HCV-induced inhibition of interferon signaling. Moreover, the addition of betaine to the standard anti-HCV therapy could overcome resistance to pegylated interferon α [13].

### 4.3. Chronic Kidney Disease (CKD)

CKD is characterized by progressive damage and a reduction in the total number of nephrons, leading to a loss of kidney function. It is caused by structural and/or functional abnormalities in the kidneys and manifested by the presence of pathohistological abnormalities and/or elevated plasma biomarkers of tissue damage, with or without a decrease in the glomerular filtration rate. Low betaine plasma levels are associated with increased kidney damage, oxidative stress, and inflammation. Therefore, betaine plasma level might be a useful biomarker for identifying CKD stages [79]. A randomized case-control study by Ephraim and Jewell [80] included 24 cats with CKD. In a group that was fed food supplemented with 0.5% betaine for 10 weeks, body composition improved, while plasma biomarkers indicated better kidney health. In another study by Ephraim and Jewell [81], a total of 28 dogs with CDK were examined. Consumption of test foods (low soluble fiber plus betaine (0.5% betaine, 0.39% oat beta-glucan, and 0.27% short-chain fructooligosaccharides) or high soluble fiber plus betaine (0.5% betaine, 0.59% oat beta-glucan, and 0.41% short-chain fructooligosaccharides)) led to a decrease in several uremic toxins. Furthermore, Sharma et al. [82] demonstrated that orally administrated betaine in rats with sodium arsenite-induced nephrotoxicity could have significant nephroprotective properties. As observed in preclinical studies, betaine has shown beneficial effects on CKD. Therefore, betaine-enriched food formulas might have the potential to be used as an additional treatment for patients with CKD [83].

### 4.4. Cardiovascular Diseases

It is well known that elevated levels of homocysteine in the blood plasma represent a risk factor for the development of cardiovascular diseases. Homocysteine is a thiol amino acid that is formed by demethylation of the essential amino acid methionine. Its adverse effects include oxidation of LDL cholesterol, increased production of collagen, reduced bioavailability of nitric oxide, and prothrombotic features. Betaine, as a methyl donor, can remethylate homocysteine, i.e., convert it to methionine [84]. According to Ashtary-Larky et al. [16], betaine supplementation at a dose of 4 g/day might have homocysteine-lowering effects. Doses of ≥4 g/day are not recommended since they can have a lipid-augmenting effect. Additionally, elevated plasma levels of homocysteine can alter the levels of apolipoprotein A1, which can cause abnormal maturation of high-density lipoprotein (HDL) particles. Keeping in mind that betaine can reduce homocysteinemia, this could normalize plasma apolipoprotein A1 levels, contributing to cardiovascular protection [85].

### 4.5. Carcinogenesis

Carcinogenesis (oncogenesis) is the process by which healthy cells are transformed into malignant cells. Currently, the associations between dietary intake of betaine and cancer risk remain obscure, mainly due to a lack of strong evidence as only a few studies are available. Van Puyvelde et al. [86] reported that betaine supplementation does not affect breast cancer risk. In a study by Lu et al. [87], no significant associations between betaine intake and lower colorectal cancer risk were observed. Additionally, in a study by Guertin et al. [88], the association between serum betaine level and colorectal cancer risk was not significant. On the contrary, Seyyedsalehi et al. [89] claim that an increased betaine intake might decrease the risk of colorectal cancer. Regarding prostate cancer, Kar et al. [90] claim that betaine has the ability to cause apoptosis and inhibit cell growth in the DU-145 human prostate cancer cell line. Han et al. [91] conducted a prospective cohort study of 6,528 men who were followed up over the years. In those with a confirmed diagnosis of prostate cancer, lethal risk was inversely associated with betaine intake. Data about betaine usage in oncology is scarce and varies to a significant extent. To overcome the lack of precision in risk estimates, more comprehensive studies are needed.

### 4.6. Neuroprotective Properties

Neuroprotection refers to different strategies and mechanisms to protect neuronal elements against damage due to acute injury or neurodegenerative disorders, such as Alzheimer’s disease. Betaine was investigated in terms of Alzheimer’s disease. In their study on HMC3 cells, Meng et al. [92] identified betaine as an autophagy inducer. The result was the clearance of amyloid-beta via the PI3K/AKT pathway. The same signaling pathway was investigated by Huang et al. [93]. In this study, betaine was able to ameliorate cognitive deficits in rats by regulating the PI3K/AKT pathway. Furthermore, it has been reported that betaine is depleted in the brains of patients with multiple sclerosis. Knowing that methionine metabolism is dysregulated in this disease, betaine, as a methyl donor, could have beneficial effects. In a study by Singhal et al. [94], it was reported that betaine can activate neuroprotective transcriptional programmers in the mouse model of multiple sclerosis. Neuroprotective agents also aim to limit inflammation and reduce oxidative damage, which is especially important in reperfusion injuries and cerebral ischemia. Li et al. [95] reported a favorable effect of betaine pretreatment in a rat model of brain infarct. Biochemical analyses revealed a decrease in pro-inflammatory cytokine production and a reduction in oxidative stress damage. Additionally, the volume of the brain infarct was reduced.

### 4.7. Body Composition and Sport Performance

Several studies on pigs and chickens in the late 1990s showed that the use of betaine could increase meat yield. Such an effect motivated researchers to look into the effects of betaine on human body composition [17]. In a double-blind, randomized, placebo-controlled trial by Cholewa et al. [96], 23 young women without prior training experience were randomly assigned to a betaine or placebo group. In a betaine group, individuals were supplemented with 2.5 g of betaine per day. After an eight-week period of structured resistance training, significant changes in lean mass and muscle thickness, as well as sports performances, were found. Nobari et al. [97] conducted another double-blind, randomized, placebo-controlled trial, which included 29 male soccer players who were randomly assigned to a betaine or placebo group. In a betaine group, individuals were supplemented with 2 g of betaine per day. After a fourteen-week period, biochemical analyses revealed increased testosterone levels in supplemented individuals, while there were no significant changes in lean body mass or body fat. According to a meta-analysis by Ashtary-Larky et al. [98], betaine supplementation does not improve body composition. No significant changes in body composition indices, such as body mass, body mass index, body fat percentage, fat mass, or fat-free mass, were noted. There are a lot of discrepant results in this field, indicating that future studies are required.

## 5. Functional Applications

For decades, betaine has been used as a feeding stimulant and feed enhancer in the nutrition of poultry, juvenile groupers, and other animal species [99,100]. This gave rise to the idea of fortifying human food with betaine. Accordingly, the quality attributes of formulated wheat flour cookies enriched with betaine at levels of 0.5 to 3% flour were investigated by Filipčev et al. [101]. Increasing betaine contents showed no significant effect on the majority of the cookie quality attributes, such as spread, height gain, hardness, and fracturability, but contributed to more prominent vividness and yellow color intensity. Sensory evaluation revealed slightly better ratings for cookies with lower fortification levels of 0.5 and 1% of flour regarding taste, total quality, and acceptability. The enriched cookies were seven-to-forty times higher in betaine in comparison to the control [101]. Afterwards, the authors fortified gluten-free biscuits prepared from corn starch and rice flour with varying levels of betaine of 0.5, 1, 2, and 3% on a flour basis [102]. The betaine content in the fortified biscuits increased four to twenty-one times in comparison to the control. Betaine addition did not cause differences in sensory attributes except for a weak aftertaste, which appeared at the highest fortification level. Textural properties did not change significantly, although the 3% betaine biscuit had somewhat lower strength. Moreover, betaine addition contributed to the yellow tone and color vividness [103]. Being consumed in large amounts on a daily basis, wheat bread was enriched with varying levels of betaine (0.68, 1.35, 2.70, and 4.05%) on a flour basis, in order to create a product that will promote dietary intake of betaine as a good practice in improving human health. At high doses, betaine affected baking performance, color, and crumb texture, thereby not substantially altering chemical composition. Fortification contributed two to twenty-nine times higher betaine contents compared to the control, depending on the formulation [100]. Betaine enriched spelt flour extruded snacks were developed by Kojić et al. [104]. Snacks were fortified with betaine in levels between 1506.8 and 1605.2 mg per portion of 40 g, taking into account that the daily portion size should be 40 g according to the recommendation of the European Breakfast Cereal Association for extruded products. Thus, the developed extruded snack products providing 1500 mg of betaine per day could provide the expected health effects.

Besides fortifying products with pure betaine, various other sources containing betaine in different amounts have been used as functional ingredients. Thus, since buckwheat has been traditionally used in the human diet for centuries, Perović et al. [105] investigated the influence of the addition of common buckwheat to wholegrain wheat flour pasta on the total betaine content. The authors obtained the highest betaine content of 21.63 mg per 100 g of product with the substitution of wholegrain wheat with 10% buckwheat flour. Gluten-free biscuits produced from corn starch and rice flour were enriched with betaine through the addition of liquid and dry sugar molasses originating from sugar beet production. With average betaine contents in the range of 5–6 g/100 g, sugar beet molasses represents a most excellent source of betaine [106]. Furthermore, the nutritional quality and betaine contents of gluten-free cookies were also enhanced by the addition of 10–30% sugar beet molasses. Molasses contributed to a significant increase in the betaine content, from 24 to over 80 times higher compared to the control, depending on the supplementation level [107]. On the other hand, Bota et al. [108] investigated the nutritional potential of spent malt rootlets, the end products of the malt industry that contain betaine and choline, as a functional baking ingredient. The study has revealed their highly desirable nutritional characteristics from a human dietary standpoint, which, in addition to their low cost and high levels of availability, make them an appropriate functional food ingredient. In recent years, beetroot (*Beta vulgaris* L.) has become popular in the context of functional food development for containing a wide range of biologically active phytonutrients, among which betaine [109]. Furthermore, a variety of algae can be useful for developing nutritional and functional foods, whether as whole algae or algal extracts. Therefore, there is a possibility of incorporating betaine into nutrition through the development of algal-based functional foods, although its content in marine macrophytes is considered very low [110,111]. As a high-quality and low-cost source of macro- and micronutrients, the fruit of a date palm is a popular, high-value commercial fruit crop in the worldwide market. A wide variety of food products with dates have been developed, including paste, syrup, juice, sugar, jam, jelly, butter, chocolates, condiments, pickled dates, oils, coffee, and others [112]. According to Musthafa and Sandhu [112], choline and its metabolite betaine are found in greater concentrations in date fruit.

In addition to the above-mentioned applications, the functional properties of betaine have been studied and exploited in various products other than food. For example, a randomized clinical trial by Rantanen et al. [113] investigated the effects of mildly flavored toothpastes with and without the addition of betaine on dry mouth, which appeared to be relieving its symptoms in patients. By showing the potential to increase exercise and sports performance, betaine has also gained attention as an ingredient in sports supplement formulations [114]. Furthermore, alkyl betaines have been used as safe functional hair and skin conditioning, antistatic, surfactant cleansing, and viscosity-increasing agents in cosmetic products [115].

Betaine-based amphiphiles, which are characterized by their zwitterionic heads and alkyl tails, have the ability to form micelles or capsules and, therefore, can be used as an agent for drug delivery. In a study by Fang et al. [116], a novel drug—a carboxybetaine conjugate—was designed by applying betaine as a hydrophilic head and antitumor drug molecules as a hydrophobic tail. The developed drug could self-assemble, forming stable nano-size vehicles with a very high drug loading. Furthermore, Han et al. [117] developed a novel zwitterionic micelle platform based on betaine polymer for oral insulin delivery. The polymeric micelles contributed to the overall enhanced intestinal absorption and high oral bioavailability of insulin.

Being an excellent protein stabilizer in various organisms and a hydrogen bond acceptor, betaine is an important component of various mixtures of deep eutectic solvents (DES) and natural deep eutectic solvents (NADES) that have found numerous applications as green alternatives in the extraction procedures of various raw materials, thus replacing organic solvents, which may have harmful effects on humans and the environment [118,119,120,121]. These are less toxic and highly biodegradable; their preparation is simple and inexpensive; and they have demonstrated a high potential for extracting the natural compounds.

## 6. Extraction and Detection in Food Matrices

According to Hefni et al. [122], there is a significant influence of extraction and separation conditions on the quantification of betaine in different food samples, emphasizing the necessity to repeat these procedures in order to obtain reliable results. Most approaches have focused mainly on conventional solid/liquid extraction, chromatography (e.g., ion exchange), and membrane technologies, but no universal method has been reported that could be applied to all food matrices.

In the study by MacKinnon et al. [123], the optimization of betaine extraction conditions from *Ascophyllum nodosum* seaweed involved the application of pure methanol and 20% solutions of water/acetone, water/methanol, and water/acetonitrile at different temperatures. The extraction with methanol at 78 °C was shown to be the most suitable. A rapid cleanup protocol and a sensitive LC–MS/MS (liquid chromatography–tandem mass spectrometry) method of analysis were developed to afford baseline separation of betaines in this brown alga. A mixture of methanol/water 40:60 (*v*/*v*) was also used to determine betaine and choline in the fractions of wheat [124]. The extractions were repeated six times, and the supernatants were analyzed by the NMR (nuclear magnetic resonance) method. Chendrimada et al. [125] quantified the betaine content in several food ingredients, including wheat. The authors investigated the extraction of betaine with a methanolic KOH solution using a Goldfisch apparatus for 3 h. Due to a number of impurities that co-eluted with betaine, HPLC with a cation exchange column (Partisil SCX-10) was used for the separation of betaine from other compounds. A cation-exchange HPLC column was also used for the separation of betaine from plant material [126]. Previously, the plant material was extracted with hot methanol or a combination of methanol/chloroform/water.

Saarinen et al. [127] investigated the betaine content in chicken liver using a Ca^2^+ cation-exchange HPLC (high-performance liquid chromatography) column with a refractive index detector, although the quantification was limited due to the low sensitivity of the detector. Considering its physical and chemical properties, this quaternary amine could not be analyzed using a conventional reverse-phase liquid chromatographic (RP-LC) system or detected with an ultraviolet (UV) detector without a prior derivatization procedure. Derivatization and analysis of betaine in numerous food products using liquid chromatography on different columns with UV detection was performed in the study by de Zwart et al. [60]. Various products grouped into 10 categories—grains, fruits, vegetables, soft drinks, alcoholic beverages, meat, seafood, dairy products, nuts, and others—were investigated in terms of betaine content [62]. Water and dichloromethane were used for extraction and 2-naptacil trifluoromethanesulfonate for derivatization. Hefni et al. [122] developed a simple HPLC–UV method for several food matrices, such as spinach, whole wheat flour, wheat, and sugar beet, whereby a derivatization procedure was carried out on a cation-exchange column.

Huang et al. [128] developed an HPLC method with an evaporative light scattering detector (ELSD) using a Luna SCX (Strong Cation Exchange) 100 Å column for the determination of betaine in *Lycii Fructus*, which was unreasonable due to the bad resolution having a very short retention time (about 5 min). Lee et al. [129] developed an ion-pair reversed-phased LC (IP-RPLC) with long perfluoropentanoic acid as the volatile ion-pairing reagent on an octadecyl silica (ODS) column and an ELSD. However, the betaine peak was not well separated from other peaks because the ion-pairing reagent of the alkyl chain could be irreversibly adsorbed to the ODS C18 stationary phase. The work of Shin et al. [130] underlined the impossibility of routine analysis of betaine in *Fructus Lycii* using the existing HPLC methods due to the presence of other amino acids that interfere with betaine quantification. Therefore, they proposed the application of a hydrophilic interaction liquid chromatography (HILIC) column in combination with ELSD using complex gradient mobile elution. Before analysis, *Fructus Lycii* samples were sonicated in an ultrasonic bath with 70% methanol for 40 min at 25 °C. To determine betaine in *Lycii Fructus*, Zhao et al. [131] developed an HPLC/ELSD method using a HILIC column with an isocratic elution. A similar approach was applied in the study by Kojić et al. [132]. The authors analyzed 54 samples of cereals and pseudocereals for betaine content by using the HPLC/ELSD method in isocratic mode. Betaine from samples was previously extracted in an ultrasonic bath for 30 min using methanol, which did not extract undesirable hydrophilic compounds and, thus, improved sample purity and increased HPLC column lifetime. In the studies by Bruce et al. [61] and Ross et al. [62], betaine cereal samples were analyzed using LC–MS/MS and an ultra-high performance liquid chromatography (UHPLC) HILIC column. An LC–MS/MS method developed by Bruce et al. [61] was applied for the analysis of 32 cereal flours and cereal fractions and 51 cereal products with previously carried out single and sequential extraction procedures, including methanol, dichloromethane, and chloroform. Ross et al. [62] analyzed a wide range of commercially available cereal foods and cereal fractions based on the previously developed extraction protocol by Bruce et al. [61]. Similarly, Spaggiari et al. [46] performed the sequential extraction of betaine and choline from cereal samples with a 50% methanol/water solution following the procedure by Bruce et al. (2010) [61], while for the separation of the analytes, a HILIC column was applied.

Being a type of normal-phase (NP) chromatography in which the stationary phase is polar, HILIC is an alternative to reversed-phase chromatography. Unlike ordinary normal-phase chromatography, larger amounts of organic solvents can be used as the mobile phase. In NP–HPLC, the mobile phases used are non-polar and do not have sufficient power to elute polar substances from the surface of the stationary phase. Consequently, the HILIC column is the most efficient column for the chromatographic separation of betaine. Thus, the HILIC type of chromatography overcomes the difficulties in the chromatographic analysis of molecules with polar characteristics. Mobile phases in HILIC systems are mixtures of water (or an aqueous buffer solution) and an organic solvent (most often acetonitrile or methanol).

Betaine has a lower absorption in the UV-visible spectrum, and therefore it is necessary to use an ELSD, which is a universal detector that provides a stable baseline, even in gradient mode, enabling the detection of the most non-volatile analytes [130]. The ELSD detector is used for compounds that are less volatile than the mobile phase and is independent of the optical properties of the compound, thus being suitable for the analysis of compounds that do not possess chromophores. Removal of the aqueous mobile phase within the ELSD is usually achieved by adjusting the temperature to the boiling point of the eluent (e.g., 100 °C) in order to remove the solvent. For non-volatile compounds, using high temperatures maximizes signal response. However, at these temperatures, volatile and semi-volatile compounds are destroyed and, therefore, not detected. This is particularly challenging for small molecules. Such detectors are, consequently, designed to evaporate highly volatile solvents at room temperature with the aim of detecting less- or non-volatile compounds. Therefore, the special technology behind the ELSD detectors reduces the evaporation time of aqueous solvents at low temperatures.

Recently, more efficient, cost-effective, and, most importantly, scalable processes replaced conventional solid/liquid extraction and chromatographic separation methods. In the study by Rivoira et al. [133], for betaine extraction from beetroot (red and gold), advanced accelerated solvent extraction (ASE) was coupled with solid-phase extraction (SPE). Two different extraction solvents were used (methanol and water–methanol, 50:50 solution) for the extraction at 50 °C. For betaine determination, a separation method based on HILIC–MS/MS and its conditions were experimentally tested, with the optimal conditions as follows: collision energy (CoE) = 40 eV; fragment voltage (FV) = 105 V; and temperature of the source = 345 °C. Recoveries were about 93%, with RSD < 5%, for both matrices, without evidence of interfering species. Moreover, a QuEChERS procedure [i.e., Quick, Easy, Cheap, Effective, Rugged, and Safe, being a type of dispersive solid-phase extraction (dSPE) used for sample preparation] was tested, achieving slightly lower recoveries than the ASE/SPE procedure (recoveries about 75%).

Mohammadzadeh et al. [134] applied the cloud point extraction (CPE) method in micelle media for the separation and recovery of betaine from beet molasses. According to Pourreza et al. [135], this simple technique enables a much higher concentration of analyte than in the case of conventional extraction because the micellar phase volume is about 10–100-fold less than the volume of an aqueous phase. The effects of pH, type and concentration of surfactant, electrolyte concentration, amount of molasses, and equilibrium time have been studied in order to establish optimum conditions. The determination of betaine concentration in samples was carried out using an HPLC–DAD method with an ODS2 C18 column. Betaine recovery from beet molasses was achieved up to 80% when three CPE steps with a total of 1.5% (*w*/*v*) of surfactant (Triton X-114) were used. Subsequently, betaine was recovered nearly 100% from the surfactant-rich phase after adjusting the pH to 2.5 and re-incubation at 40 °C. Abdollahzadeh et al. [136] performed the extraction, recovery, and purification of betaine from sugar beet molasses based on the optimal conditions proposed in the previously mentioned study. According to the HPLC chromatogram results of cloud point extraction, the recovery rate of betaine from sugar beet molasses using this method was 81.32%. The recovery of betaine from aqueous betaine and sugar beet industry by-products (vinasse and molasses) solutions by reactive extraction was studied by Altinisik et al. [8]. Dinonylnaphthalenedisulfonic acid (DNNDSA) dissolved in four different organic solvents (toluene, dimethyl phthalate, 1-octanol, and methyl isobutyl ketone) to form organic phases, was used as the extraction agent. Moreover, the effects of various process parameters such as the DNNDSA concentration (0.1–0.5 M), temperature (298–338 K), and pH (2.0–12.0) on the extraction efficiency were investigated. HPLC–UV analysis indicated that higher DNNDSA concentration and temperature with toluene as an organic phase solvent recovered ~71.5%, 71%, and 67.5% of the betaine in vinasse and molasses solutions, respectively.

Overall, the recovery of betaine from natural sources is of great interest but also challenging. In order to overcome the cost of purchasing, operating, and maintaining expensive separation means, new extraction and separation techniques were developed and preferred due to their high efficiency, selectivity, operational practicality, and low cost and energy demand. The HILIC–MS/MS method proved to be able to analyze betaine in different food samples. With a relatively rapid and simple sample preparation and short runtime, this method could be faster and more efficient than most existing methods used for food analysis.

## 7. Conclusions and Perspectives

Betaine plays three major roles in the mammalian organism. Firstly, as an organic osmolyte, it maintains normal cell volume under osmotic stress caused by various factors. Secondly, it provides protection against protein denaturation, and, thirdly, besides methylfolate, betaine is the only molecule that provides methyl groups for homocysteine remethylation.

Food that contains either betaine or choline provides the body with betaine. Betaine is not regarded as an essential food because it may be irreversibly produced in the human body from free choline with the aid of the choline dehydrogenase enzyme. However, the body’s natural ability to produce betaine is typically insufficient to meet daily requirements. Therefore, it may be said that consuming betaine through diet is required. The highest concentrations in food matrices were observed in cereal grains, pseudocereals (especially amaranth and quinoa), cereal products (wholegrain flour, bread, pasta, couscous, and breakfast cereals), some vegetables (spinach and beetroot), and the majority of seashells (mussels, oysters, clams, and scallops).

Many pre-clinical studies gave good insights into the possible antioxidant, hepato-, nefro-, cardio-, and neuroprotective roles of betaine supplementation, as well as its ability to enhance endurance and sports performance. Nevertheless, to confirm or reject the results of the previously published pre-clinical investigations, more randomized, placebo-controlled trials are inevitable.

In the nutrition of chicken, young grouper, and other animal species, betaine has been utilized for many years as a feeding stimulant and feed enhancer. It was this that inspired the concept of a betaine-fortified human diet. However, only a few studies have explored the potential of betaine as a functional food ingredient, thereby leaving a gap for further research in this field. Furthermore, in vitro and in vivo investigations are necessary in order to investigate the betaine digestion process in humans and animals.

New advanced extraction techniques can be proposed as efficient, clean, and simple separation techniques for use in betaine recovery from different food matrices instead of the expensive, sophisticated, and time-consuming chromatography process. Further, the HILIC–MS/MS method may provide fast and precise identification and quantification of betaine, finding relevant applications in the food, pharmaceutical, and agricultural fields.

## Figures and Tables

**Table 1:** Reported betaine contents in various food and beverage sources.

Food Source	µg/g DW	Ref.	Food Source	µg/g DW	Ref.
Cereals, Pseudocereals, and Products			Cereals, Pseudocereals, and Products		
Flour (wheat, refined)	141–718	[58,61]	Noodles (egg)	190–1300	[58,62]
Flour (wheat, wholegrain)	604–1503	[58,61]	Biscuits	160–430	[63]
Flour (spelt, refined)	410–978	[58]	Crackers (refined)	401–460	[61]
Flour (spelt, wholegrain)	1296–1442	[58]	Crackers (wholegrain)	694–920	[61]
Flour (rye, refined)	310	[58]	Crackers (rice)	6	[63]
Flour (rye, wholegrain)	986–1500	[58]	Bran (wheat)	2300–7200	[58,61,63]
Flour (barley, refined)	250	[58]	Bran (rye)	1650–2135	[61]
Flour (barley, wholegrain)	776–1023	[58]	Bran (oat)	188	[61]
Flour (corn, refined)	2	[58]	Grains (wheat)	490–1320	[58]
Flour (corn, wholegrain)	120	[58]	Grains (spelt wheat)	565–2723	[58]
Flour (rice, refined)	8	[58]	Grains (triticale)	986–1030	[58]
Flour (amaranth, wholegrain)	871–1225	[58]	Grains (rye)	444–2213	[58]
Flour (proso millet, refined)	1320	[58]	Grains (oat)	200–1000	[58,60]
Flour (buckwheat, wholegrain)	7–108	[58]	Grains (barley)	460	[58]
Flour (sorghum, refined)	425	[58]	Grains (corn)	5–304	[58,60]
Bread (white wheat)	174–520	[58,61,62,63]	Grains (proso millet, dehulled)	281	[58]
Bread (wholegrain wheat)	499–1000	[58,60,62,63]	Grains (quinoa)	610–6300	[58,62]
Bread (wholegrain rye)	855–1666	[58,61]	Grains (buckwheat)	6–26	[58,61]
Bread (wholegrain spelt)	913	[58]	Grains (amaranth)	646–7420	[58]
Pasta (white wheat)	222–773	[58,61]	Grains (white rice)	2–5	[61,62]
Pasta (wholegrain wheat)	375–1327	[58,61,62]	Grains (brown rice)	3–9	[61,62]
Pasta (barley)	211	[58]	Corn flakes	6–120	[58,63]
Pasta (cooked)	228–352	[60]	Flips (white, small grains)	100–200	[62]
Couscous (wholegrain)	544–1299	[61]	Biscuits	160–430	[63]
Tortilla (wheat)	311	[58]	Fruit and Vegetables		
Breakfast cereal	10–1041	[61]			
Noodles (white rice)	3	[61]	Apricot	Trace	[62]
Noodles (brown rice)	6	[61]	Apples	1	[57]
Grapes	1	[57,63]	Legumes and Products		
Oranges	1	[57]			
Blueberries	2	[57]	Peanuts	6	[57]
Strawberry	2	[57,63]	Soybean	21	[57,59]
Grapefruit	2	[57]	Tofu	4	[57]
Peaches	3	[57]	Lentils	<10	[60]
Watermelon	3	[57]	Peas	<5	[60]
Prune	4	[57,59,60]	Oils and Fats		
Banana	<5	[60]			
Kiwifruit	<5	[60]	Margarine (olive oil)	<5	[60]
Pear	<10	[60]	Olive oil	1	[57]
Avocado	3–35	[57,59,60,63]	Fungi		
Raisins	3	[57,59]			
Tomato	Trace	[63]	Mushrooms	10–110	[57,59,60]
Onion	Trace	[60,63]	Spices and Herbs		
Celery	1	[57]			
Potato	26–350	[59,63]	Pepper (red)	12	[63]
Asparagus	33–45	[63]	Pepper (green)	24–31	[63]
Cabbage	3	[57,59]	Mustard seed	19	[57]
Zucchini	3	[57]	Meat and Seafood		
Carrot	4	[57,59]			
Broccoli	<10	[60]	Beef	58–170	[59,60,63]
Cauliflower	<10	[60]	Mutton	62–180	[63]
Garlic	<10	[60]	Lamb	72	[60]
Lettuce	<10	[60]	Pork	41	[60]
Beetroot	750–1290	[57,60]	Chicken breast fillet	180–200	[59,60]
Beetroot (canned)	2600–3337	[57,59]	Chicken liver	129	[57,59]
Silverbeet	910	[60]	Bacon	20–97	[57,59,63]
Spinach	675–1100	[57,59,60]	Ham	81–95	[63]
Sausage (beef, raw)	320	[63]	Beverages		
Sausage (pork)	36	[59]			
Fish (fresh)	120–150	[63]	Orange juice	3–20	[57,59]
Fish (canned)	20–45	[63]	Apple juice	1	[57]
Fish sticks	330	[59]	Coffee (instant)	62–68	[63]
Clams	2500	[59,60]	Tea (black)	10–120	[59,60,63]
Cod	25	[60]	Wine (white)	Trace	[63]
Groper	12	[60]	Wine (red)	<5	[60]
Monkfish	500	[60]	Beer	56–81	[59,63]
Mussel	1120–11,600	[60]	Ready-to-eat Food		
Oyster	2780–2810	[60]			
Scallops	640–1180	[60]	Pizza	260	[57,59]
Salmon	19–43	[59,60]	Hamburger	333	[57,59]
Perch	26	[59,60]	Hot dog and bun	443	[57,59]
Tuna	<10	[60]	Fat-free salad dressing	18	[59]
Dairy and Eggs			Tacos/burritos	150	[57]
			Lasagna	61	[57]
Milk	7–28	[57,59,60,63]	Falafel	202	[60]
Milk (chocolate)	6	[63]	Milk chocolate	3–26	[57,59,60]
Milk (soya)	12	[63]	Doughnuts	270–380	[58]
Yoghurt	5–9	[57,59,60]	Apple pie	160	[57,58]
Sour cream	5–7	[57,60]	Danish pastry	140	[57,58]
Cheese	5–67	[57,59,60,63]	Ready-to-eat pancakes	690–720	[58]
Ice cream	6–11	[57,59]	Fat-free salad dressing	18	[59]
Butter	3	[57]	Soy sauce	396	[57,59]
Eggs	10–60	[57,59,60]	Popcorn	4	[57]

## Data Availability

Data are available from the corresponding author upon request.
